# Mediterranean spotted fever-like illness caused by *Rickettsia sibirica mongolitimonae*, North Macedonia, June 2022

**DOI:** 10.2807/1560-7917.ES.2022.27.42.2200735

**Published:** 2022-10-20

**Authors:** Dejan Jakimovski, Sofija Mateska, Verica Simin, Ivana Bogdan, Dragana Mijatović, Agustín Estrada-Peña, Lourdes Mateos-Hernández, Angélique Foucault-Simonin, Sara Moutailler, Alejandro Cabezas-Cruz, Pavle Banović

**Affiliations:** 1 University Clinic for Infectious Diseases and Febrile Conditions, Skopje, North Macedonia; 2 Faculty of Medicine, Ss. Cyril and Methodius University in Skopje, Skopje, North Macedonia; 3 Department for Microbiological and Other Diagnostics, Pasteur Institute Novi Sad, Novi Sad, Serbia; 4 Ambulance for Lyme Borreliosis and Other Tick-Borne Diseases, Department of Prevention of Rabies and Other Infectious Diseases, Pasteur Institute Novi Sad, Novi Sad, Serbia; 5 Department of Animal Health, Faculty of Veterinary Medicine, University of Zaragoza, Zaragoza, Spain; 6 Research Group in Emerging Zoonoses, Instituto Agroalimentario de Aragón-IA2 (Universidad de Zaragoza-CITA), Zaragoza, Spain; 7 ANSES, INRAE, Ecole Nationale Vétérinaire d’Alfort, UMR BIPAR, Laboratoire de Santé Animale, Maisons-Alfort, France; 8 Department of Microbiology with Parasitology and Immunology, Faculty of Medicine in Novi Sad, University of Novi Sad, Novi Sad, Serbia; * These authors contributed equally

**Keywords:** *Rickettsia sibirica mongolitimonae*, *Hyalomma*, North Macedonia, Mediterranean spotted fever-like illness

## Abstract

Mediterranean spotted fever-like illness (MSF-like illness) is a tick-borne disease caused by *Rickettsia sibirica mongolitimonae* first reported in France more than 25 years ago. Until today, more than 50 cases of MSF-like illness have been reported in different regions of Europe and Africa, highlighting variable clinical manifestation. Here we report a case of MSF-like illness following a bite from a *Hyalomma* tick in the Skopje region of North Macedonia.

Mediterranean spotted fever (MSF)-like illness is tick-borne disease caused by *Rickettsia sibirica mongolitimonae,* an obligate intracellular bacterium, most probably transmitted by ticks of the genus *Rhipicephalus* and genus *Hyalomma* [1]. While cases of MSF, which are caused by *Rickettsia conorii*, are frequently detected in endemic areas of Europe around the Mediterranean Basin, MSF-like illness is reported sporadically in different European and African countries [1,2]. Here we present a case of MSF-like illness caused by *R. sibirica mongolitimonae* in a female patient after a bite from a *Hyalomma* tick in North Macedonia.

## Case description

**Figure 1:**
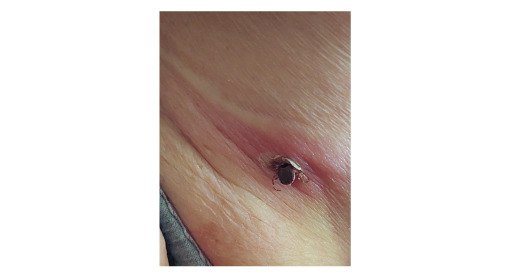
*Hyalomma* tick attached to the skin of left axillar region with circumscriptive erythematous patch around the infestation site, North Macedonia, June 2022

**Figure 2:**
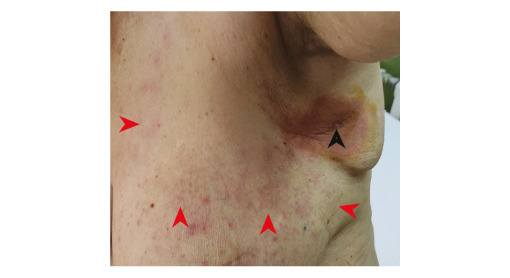
Clinical presentation of *Rickettsia sibirica mongolitimonae* infection (6th day of illness), North Macedonia, June 2022

A female patient in her 60s visited an outpatient department of our clinic in Skopje, North Macedonia in June 2022. The patient reported a rash accompanied with myalgia, headache, fever (38.5 °C) and malaise. Symptoms had started 6 days earlier (on 15 June, Day 1) with high-grade fever, headache, myalgia and malaise. Three days later (Day 4), she noticed a tick surrounded with a circumscriptive erythematous patch under her left axilla (Figure 1). The patient visited the emergency department, where the tick was surgically removed, and antibiotic treatment (amoxicillin/clavulanic acid 1,000 mg twice a day) was administered. On Day 7 (the day she visited our clinic), a non-pruritic rash was noticed on both breasts and upper arms (Figure 2) and non-specific symptoms persisted despite antibiotic treatment. 

Black arrow: location of tick infestation. Red arrows: skin affected by maculopapular rash caused by infection.

The patient lived in a house in a rural area of the Skopje region in North Macedonia. She reported that she had not travelled outside that region in the previous 6 months. In the context of possible contact with animals, the patient reported that she only had a pet dog. She declined a household visit and blood sampling of the animal, therefore exposure of the dog to tick-borne pathogens could not be assessed.

During the consultation on Day 7, we also took a venous blood sample for biochemical analysis and complete blood count. A capillary blood sample from the tick infestation site and another venous blood sample were taken for direct detection of spotted fever group rickettsiosis (SFGR). A treatment regimen including doxycycline 100 mg twice daily for 14 days was started without awaiting the test results, and the patient was managed through the outpatient department. Control examination was conducted on Day 9, where the patient showed an excellent clinical response. The rash stopped spreading immediately after the antibiotic regimen was changed to tetracyclines, while the fever receded on the second day of the treatment. After 10 days of doxycycline treatment, another control examination was conducted. The patient reported no complaints, while hyperpigmentation was noticed at the previous skin lesions and tick infestation site.

In order to confirm the exposure to an SFGR member, a serum sample was taken 4 weeks after the symptom onset for examination of seroreactivity against *R. conorii* antigens.

## Laboratory findings

### Complete blood count and biochemical analysis

Complete blood count was performed on an automated hematology analyzer XS-1000i (Sysmex, Kobe, Japan). All biochemical analyses quantifying levels of creatinine, aspartate aminotransferase (AST), alanine transaminase (ALT) and C-reactive protein (CRP) were performed on a Cobas Integra 400 Plus II analyzer (Roche Diagnostics Corporation, Indianapolis, United States (US)). Among the initial laboratory findings, only C-reactive protein showed mild elevation (39 mg/L; reference: 0–10 mg/L). Complete blood count, AST, ALT and creatinine values were unremarkable (Table).

**Table:** Patient data, disease manifestation and laboratory findings, *Rickettsia sibirica mongolitimonae* infection, North Macedonia, June 2022

	Case	
**General data**		
Patient age group (years)/sex	60–70/female	
Date of illness onset	16 June 2022	
Tick bite reported	18 June 2022	
Reported location of tick encounter	Skopje region, North Macedonia	
Tick genus	*Hyalomma*	
**Clinical manifestation** (yes/no)		
Fever	Yes	
Headache	Yes	
Myalgia	Yes	
Eschar	Yes	
Enlarged regional lymph nodes	No	
Local redness	Yes	
Systemic rash	Yes	
Outcome	Recovered	
Complications	Hyperpigmentation of skin affected by infection	
**Laboratory findings**		
White blood cells	8.1 × 10^3^ µL	Reference: 4.0–11.0 × 10^3^ µL
Platelets	245 × 10^3^ µL	Reference: 150–400 × 10^3^ µL
AST	34 U/L	Reference: 10–47 U/L
ALT	35 U/L	Reference: 10–52 U/L
CRP	39 mg/L	Reference: 0–10 mg/L
Creatinine	83 µmol/L	Reference: 62–133 µmol/L
IgG against SFGR	+ (1:512)	Negative (< 1:64)

ALT: alanine transaminase; AST: aspartate transaminase; CRP: C-reactive protein; SFGR: spotted fever group rickettsiosis.

### Morphological characterisation of the tick

Although the tick was not submitted for entomological examination, the patient provided a photography of the still-attached tick before removal at the emergency department (Figure 1). It was taxonomically identified as member of the genus *Hyalomma* according to taxonomic keys described previously [3].

### Direct detection of the causative agent

We extracted total DNA from complete blood and the capillary blood sample using the NucleoSpin Tissue kit (Macherey Nagel, Düren, Germany). Initially, was assessed the presence of SFGR DNA with a probe-specific RealLine Rickettsia Species Fla-Format kit (Bioron Diagnostics, Römerberg, Germany; catalogue number VBD5392). The qPCR reaction was performed using the StepOne Real-Time PCR System (Applied Biosystems, San Francisco, US). From the two blood samples acquired from the patient, *Rickettsia* spp. DNA was detected only in capillary blood.

Subsequently, total DNA extracted from the capillary blood sample was analysed via high-throughput microfluidic real-time PCR amplification using 48.48 Dynamic Array IFC chips (Fluidigm, San Francisco, US) as described previously [4]. The capillary blood sample tested positive in the microfluidic PCR for *Rickettsia* spp. *gltA* gene. Sequencing of *gltA*, *ompA* and *ompB* gene fragments revealed the presence of *R. sibirica mongolitimonae* (GenBank accession numbers OP425002, OP515622 and OP605957, respectively). See the Supplement for details concerning validation of microfluidic real-time PCR and amplicon sequencing.

### Indirect immunofluorescence analysis

Since antibodies against one SFGR member cross-react with antigens of all SFGR members [5], we examined exposure to tick-borne rickettsial pathogens via detection of IgG reactive with *R. conorii* using a commercial indirect immunofluorescence assay (Vircell, Granada, Spain; reference ‘PRICOG’). We examined twofold serial serum dilutions, starting from 1:32 up to 1:1,024. The serum sample acquired from the patient 4 weeks after the first medical examination tested positive for anti-SFGR IgG and showed seroreactivity up to dilution 1:512, suggesting recent infection with an SFGR member. The serum sample acquired during the first visit to our clinic was reactive only in dilution 1:32.

## Discussion

MSF-like illness, caused by *R. sibirica mongolitimonae* infection, was previously described as an emerging disease in France, Portugal, Spain, Turkey and several African countries [1,2,6]. The illness probably occurs in a wider geographical area, since *R. sibirica mongolitimonae* was also detected in ticks from Israel and China [7,8]. On the Balkan peninsula, Turkey is the only country with previous reports of MSF-like illness [1], while MSF cases have been reported in Bulgaria, Croatia, Greece, Romania and Turkey [9-13]. Although MSF is not reported in Serbia, recent reports of tick-borne lymphadenopathy [14] and atypical clinical manifestations caused by *Rickettsia helvetica* [4] indicate circulation of SFGR in the local population.

Here, we report the first case of MSF-like illness detected in North Macedonia, caused by *R. sibirica mongolitimonae* and transmitted by a *Hyalomma* tick. According to the classification proposed for evaluation of MSF clinical manifestation [15], our case had a mild, typical and acute form of disease, given that the patient developed fever, headache, myalgia, eschar and a systemic rash, with elevated CRP levels as the only abnormal laboratory finding. Currently ticks of the genera *Rhipicephalus* and *Hyalomma* are proposed as the main vectors for *R. sibirica mongolitimonae* [1].

Earlier studies in North Macedonia noted the presence of *Hyalomma* and *Rhipicephalus* ticks in the Skopje region, the South-eastern region and the North-eastern region [16]. In addition, their presence was confirmed indirectly in Vardar region [16]. This report of human exposure to *Hyalomma* ticks are in accordance with previously published data, as the household of our patient is located within Skopje region and in near proximity to Vardar region.

Diagnostics of MSF-like illness require an experienced clinician who will consider *R. sibirica mongolitimonae* infection as a differential diagnosis and further require the existence of a highly specialised microbiological laboratory facility which is able to confirm the rickettsial aetiology of the disease. In addition, MSF-like illness presents a diagnostic challenge since it can appear during winter and can have variable clinical manifestations [1,17].

## Conclusion

Considering that MSF or MSF-like illness are not nationally notifiable diseases in North Macedonia, there is a high possibility that MSF-like illness is neglected, especially in North Macedonian regions where presence of *Hyalomma* and *Rhipicephalus* ticks is reported. Since precise aetiological diagnosis of MSF-like illness requires a highly specialised microbiological laboratory facility, we are proposing cross-border cooperation of Balkan countries to standardise the diagnostic procedure and raise awareness among physicians, building diagnostic capacity and networks in which information about cases can be shared. This will allow to plan effective preventive measures and to assess the impact of this disease in the population of North Macedonia and as well as population of other Balkan countries possibly affected by *R. sibirica mongolitimonae* infection. 

## Supplementary Data

166000 Supplement
