# Supplementary material for: Mediterranean spotted fever-like illness caused by Rickettsia sibirica mongolitimonae, North Macedonia, June 2022
Source: Euro Surveill. 2022 Oct 20;27(42):2200735. doi: 10.2807/1560-7917.ES.2022.27.42.2200735 (PMC9585876; doi:10.2807/1560-7917.ES.2022.27.42.2200735)
Supplement: Supplement [file 22-00735_BANOVIC_Supplement.pdf]

Supplement

This supplementary material is hosted by *Eurosurveillance* as supporting information alongside the article *Mediterranean spotted fever-like illness caused by Rickettsia sibirica mongolitimonae, North Macedonia, June 2022*, on behalf of the authors, who remain responsible for the accuracy and appropriateness of the content. The same standards for ethics, copyright, attributions and permissions as for the article apply. Supplements are not edited by *Eurosurveillance* and the journal is not responsible for the maintenance of any links or email addresses provided therein.

Validation of microfluidic real-time PCR and amplicon sequencing

In order to validate real-time microfluidic PCR results, the *gltA*-positive sample was subjected to further conventional PCR assays using the primers Rsfg877/Rsfg1258 (1), Rr190.70p/Rr190.602n (2), and Rc.rompB.4,496p/Rc.rompB.4,762n (3) which target fragments of the rickettsial genes *gltA*, *ompA*, and *ompB*, respectively.

Amplicon sequencing was commissioned to Eurofins MWG Operon (Ebersberg, Germany) and sequences were assembled using the BioEdit software (Ibis Biosciences, Carlsbad, CA, United States). The final nucleotide sequences were analyzed to identify the sequenced microorganisms using the GenBank database through the Basic Local Alignment Sequence Tool (BLAST).

Obtained nucleotide fragments of *gltA* (accession number OP425002), *ompA* (accession number OP515622), and *ompB* (accession number OP605957) genes showed high identity (>99.5%) with *Rickettsia sibirica mongolitimonae* sequences available in GenBank.

References:

1. Regnery, R. L., Spruill, C. L., and Plikaytis, B. D. (1991). Genotypic identification of rickettsiae and estimation of intraspecies sequence divergence for portions of two rickettsial genes. *J. Bacteriol.* 173, 1576–1589. doi: 10.1128/jb.173.5.1576-1589.1991
2. Robson F C Almeida, Marcos V Garcia, Rodrigo C Cunha, Jaqueline Matias, Marcelo B Labruna, Renato Andreotti. The first report of Rickettsia spp. in Amblyomma nodosum in the State of Mato Grosso do Sul, Brazil. Ticks Tick Borne Dis. 2013 Feb;4(1-2):156-9. doi: 10.1016/j.ttbdis.2012.08.002.
3. Yeon-Joo Choi 1 , Seung-Hyun Lee, Kyung-Hee Park, Young-Sang Koh, Keun-Hwa Lee, Hyung-Suk Baik, Myung-Sik Choi, Ik-Sang Kim, Won-Jong Jang. Evaluation of PCR-based assay for diagnosis of spotted fever group rickettsiosis in human serum samples. Clin Diagn Lab Immunol. 2005 Jun;12(6):759-63. doi: 10.1128/CDLI.12.6.759-763.2005.
